# Stability Preparedness: The Not-So-Cold Case for Innovations in Vaccine Stability Modelling and Product Release

**DOI:** 10.3390/vaccines12091000

**Published:** 2024-09-01

**Authors:** Franz Schnetzinger, Didier Clénet, Philippe-Alexandre Gilbert, Antonio Guzzi, Marilena Paludi, Jos Weusten, Renske Hesselink

**Affiliations:** 1 Manufacturing & Supply Chain, Coalition for Epidemic Preparedness Innovations, Askekroken 11, 0277 Oslo, Norway; 2 Global Bioprocess Development, Vaccine CMC Development & Supply, Sanofi, 1541 Avenue Marcel Merieux, 69280 Marcy-l’Étoile, France; didier.clenet@sanofi.com; 3 Gates Foundation, Seattle, 500 5th Ave N, Seattle, WA 98109, USA; philippe-alexandre.gilbert@gatesfoundation.org; 4 Regulatory Affairs, Coalition for Epidemic Preparedness Innovations, 215 Euston Road, London NW1 2BE, UK; antonio.guzzi@cepi.net; 5 GSK Vaccines Srl, Technical Research and Development, Via Fiorentina 1, 53100 Siena, Italy; marilena.x.paludi@gsk.com; 6 MSD, Center for Mathematical Sciences, Vollenhovermeer 2, 5347 JV Oss, The Netherlands; jos.weusten@merck.com

**Keywords:** pandemic preparedness, thermostability, vaccine platform, stability modelling, labelling

## Abstract

The rapid development of equitably accessible vaccines is paramount in addressing emerging global health challenges. The safety and efficacy of vaccines hinge significantly on their ability to remain stable from manufacturing throughout the supply chain and up to administration. Furthermore, the release of vaccines requires sufficient understanding of the stability profile to allow for expiration dating. In the event of a public health crisis, the time to generate the necessary stability data and the need for rapid product release are in direct opposition. Developing manufacturing platforms with thermostable product formulations for rapid response is therefore key to meeting CEPI’s 100 Days Mission goal. This Review aims to highlight the need for stability preparedness through developing thermostable vaccine platforms and exploring innovative stability monitoring strategies that leverage advanced technologies, predictive modelling, and adaptive methodologies. By doing so, we seek to enhance the efficiency and effectiveness of stability assessments, supporting rapid development, regulatory approval, and widespread, equal distribution of vaccines—especially in an outbreak scenario. Finally, enhanced thermostability will allow for simplification across the supply chain, which will reduce the financial burden of vaccination programmes and enhance equitable access.

## 1. Introduction

The Coalition for Epidemic Preparedness Innovations (CEPI) and others announced the 100 Days Mission [1] as an ambitious global goal to respond more rapidly to the next pandemic threat, to make vaccines ready for initial authorization and manufacturing at scale within 100 days of recognition of a pandemic pathogen. To meet this aspiration, a fundamental shift towards preparedness is required in almost all areas of vaccine development, manufacturing, and distribution [2]. In the research exercise conducted by CEPI, the need for improved thermostability has been identified to allow easier formulation and speed up distribution for equitable access. Furthermore, the use of agile labelling systems is discussed as a prerequisite for flexible shelf-life adjustment as additional data are gathered, allowing the regulatory approval of products without the need for extensive stability studies upfront.

This Review examines the scientific and regulatory advances which can support the introduction of thermostable vaccines and a rolling shelf-life review. This paper lays out a future state in which a combination of multiple stability-related innovations allows for significant acceleration in the development and deployment of new vaccines to combat disease outbreaks. Specifically, we have identified three areas of investment to address the challenges posed by the 100 Days Mission and to make rapid response vaccines more equitably accessible:

1. rapid response vaccine manufacturing platforms, formulations, and/or novel delivery systems with improved thermostability;
2. methods to rapidly test and predict stability of a new vaccine candidate through leveraging prior knowledge of a manufacturing platform and stability modelling;
3. labelling systems to allow flexible update of expiry dates as evidence becomes available.

**Figure 1:**
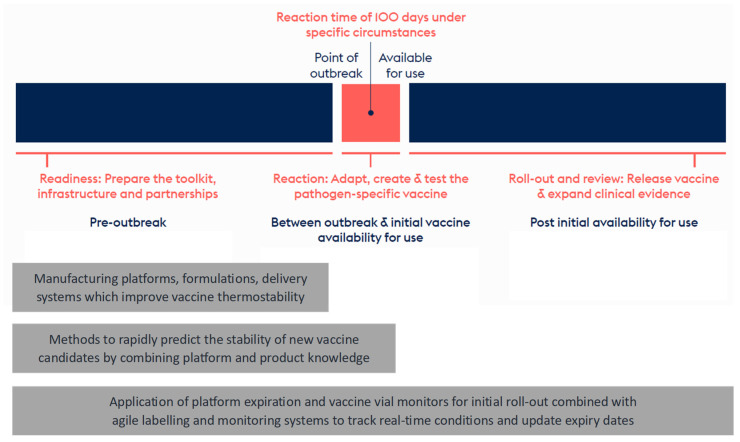
Innovations in thermostable vaccines are key to raise manufacturing readiness, allow quick reaction in case of an outbreak, and reduce supply chain complexity and vaccine waste during roll-out.

The three innovation areas include scientific and technical improvements to vaccine thermostability and stability evaluation. In addition, updated regulatory guidelines and pathways to reduce the time for vaccine release in rapid response scenarios are considered key in reducing vaccine development timelines. Deployment of these innovations will increase preparedness for future outbreaks and is expected to significantly contribute towards the 100 Days Mission (Figure 1).

Because of the interlinked nature of these innovations, close collaboration and alignment across product developers, industry partners, and health authorities will be essential for successful deployment. Incremental improvements in each innovation area are expected to lead to a reduction of vaccine development timelines. However, to address the gaps identified in this report and to achieve a step-change in how vaccine stability is evaluated and monitored, a concerted effort by all stakeholders is required.

The focus of this Review is a rapid response situation in case of an epidemic or pandemic outbreak. Nevertheless, the tools presented in the report can be used for more efficient vaccine development and deployment in non-outbreak situations. This paper encompasses all vaccine manufacturing platforms. The stability, shelf life, and labelling considerations provided in this paper are applicable to liquid and lyophilized formulations, including adjuvanted products. Furthermore, the topics discussed herein apply to novel presentations such as patches, microfilms, and solid dosage forms. (In this Review the term thermostable vaccine is used for all innovations leading to enhanced stability of vaccine products).

## 2. Problem Statement

Great progress has been made in vaccine research and development since the COVID-19 pandemic began in late 2019. Vaccines have been developed and licensed much more rapidly than ever before [1], using both traditional approaches such as inactivated pathogen vaccines as well as novel technologies including mRNA-, viral vector-, and recombinant protein-based vaccines [3]. mRNA technology in particular made its breakthrough in 2020, with the first licensed mRNA vaccines for COVID-19 after decades of development [4].

And yet, despite profound improvements in vaccine development and manufacturing, the release and distribution of vaccines is still heavily driven by factors concerning product stability. Although each class of vaccine requires specific considerations, degradation is typically associated with external factors such as heat, light, radiation, and agitation [5]. Furthermore, reactions with the container or formulation components may lead to vaccine instability presented as a loss in potency and safety.

Appropriate product design following quality by design principles may mitigate some factors which can cause accelerated product degradation. Nevertheless, vaccine development typically requires significant effort to evaluate product stability. This includes but is not limited to optimization of product formulation and studies at accelerated and stressed conditions to characterize the impact of external factors. Learnings from vaccination programmes indicate that temperature is often the highest contributor to product degradation and the most difficult to control in the supply chain [6]. This, in turn, drives the need for extensive stability studies upfront to assign a product expiration date. In addition, to minimize the potential impact of temperature excursions, specialized shipping and storage infrastructure, together with the necessary procedures and records, are needed [7]. All of which complicate distribution and delivery, particularly in remote regions, and add to the cost of immunization. Overall, a significant financial burden and waste in vaccine doses can be attributed to inappropriate storage conditions during shipment and storage [8,9].

The development of thermostable vaccines through novel formulations or more stable presentations/delivery mechanisms is key in reducing the impact of temperature variations or different climatic regions on the supply chain infrastructure. To release thermostable vaccines in a rapid response scenario, the evaluation of product stability is seen as a critical path in drug substance and drug product manufacturing. Traditionally, long-term stability studies at the storage condition, supported by accelerated and stressed stability data, inform the expiration date. When vaccines are developed rapidly in response to an epidemic or pandemic, there is limited time to perform long-term stability studies on that specific vaccine. This leads to a short initial shelf life for a new product, which, in turn, further complicates the already challenging distribution and delivery to remote regions. Additionally, using expiry dates for labelling makes it difficult to update the shelf life as additional data are generated. While vaccines are typically used within a relatively short time in outbreak situations because of the urgent need, a minimal shelf life of ca. 6 months is still required for release, distribution, and delivery, especially in remote regions. This is challenging to achieve in a 100 Days Mission scenario. In addition, the innovations described here will enable more efficient and development and deployment of equitably accessible vaccines in non-outbreak situations. 

To advance stability assessment, the combination of multiple stability related innovations will be required. It is expected that addressing each challenge individually will lead to incremental improvements in stability preparedness. However, a holistic approach towards stability preparedness will result in a more significant contribution to creating a new paradigm for rapid vaccine response.

## 3. The Need for Thermostable Vaccines

The urgent need for thermostable vaccines became clear during the COVID-19 pandemic. Of the over 13.6 billion doses of COVID-19 vaccines delivered, 39% have been administered in LIC+LMIC regions and 61% in HIC+UMIC regions [10] (official data collated by Our World in Data—processed by Our World in Data. “Total vaccinations” [COVID-19 dataset as of 29 December 2023; accessed 13 June 2024]). While this appears to be a success in terms of vaccine equity, the types of COVID-19 vaccines that were available differs strongly by region. In HIC+UMIC, the majority of total doses delivered were mRNA-based vaccines, whereas other platforms dominated in LIC+LMIC [11,12]. The mRNA-based vaccines offered major advantages during the COVID-19 pandemic, including early availability, high efficacy, and a favourable safety profile. The platform is currently being further developed for multiple existing as well as novel threats, and global access to mRNA-based vaccines is therefore essential for equity.

While there were multiple reasons for the inequitable distribution of mRNA-based vaccines, including political and financial ones [13], the requirement for (ultra)cold storage was certainly a contributing factor [14]. Comirnaty^®^ (Pfizer/BioNTech COVID-19 vaccine, New York, NY, USA, and Mainz, Germany) has a real-time storage temperature between −90 °C and −60 °C (ultracold freezer), while for Spikevax^®^ (Moderna, Cambridge, MA, USA) this is between −50 °C and −15 °C. Short-term storage at 2–8 °C is currently allowed for 10 weeks for Comirnaty^®^ and for 30 days for Spikevax^®^, up from the initial 5 and 7 days, respectively. Vaccines based on other platforms, such as viral vector- and protein-based vaccines, can be stored long-term in a well-established refrigerated cold chain between 2 °C and 8 °C, which greatly facilitates distribution. Already prior to the COVID-19 pandemic, improved thermostability was the most desired vaccine attribute for outreach and campaign settings in a study conducted by VIPS including immunization stakeholders in 61 countries [15]. The positive impacts of thermostable vaccines are summarized in Table 1.

It is thus clear that vaccine thermostability improvements are essential to achieve equitable access—in particular for platforms that currently require frozen storage such as mRNA but also, for example, the vesicular stomatitis virus (VSV) platform used for, e.g., MSD’s Ebola vaccine VSV-EBOV. The inherent properties of mRNA as well as the lipid nanoparticle carrier (LNP) make them sensitive to thermal and other stresses [16]. Various approaches are being investigated to enhance mRNA vaccine stability, including different carrier systems, optimization of pH and stabilizing excipients, process improvements, lyophilization, or novel formulation technologies such as microarray patches and solid dosage forms [17]. To advance these important innovations, CEPI published a Call for Proposals in January 2022 focused on vaccine thermostability and included the topic again in the Call for Proposals ‘Innovations to Prepare for Future Epidemics and Pandemics’, published online (available online: https://cepi.net/calls-for-proposals (accessed on 30 July 2024)) in October 2023. These initiatives have resulted in a number of promising development projects working towards thermostable RNA-based vaccines (see, e.g., available online: https://cepi.net/coming-cold-needle-free-patch-technology-mrna-vaccines-aims-end-need-frozen-storage-and-improve, https://cepi.net/stabilised-mrna-vaccine-technology-aims-end-need-frozen-storage-and-improve-access, https://cepi.net/needle-free-vaccine-delivery-platform-aims-end-frozen-storage-needs-and-improve-access, https://cepi.net/cepi-partners-biotech-jurata-thin-film-create-needle-free-mrna-vaccines-and-improve-access and https://cepi.net/new-spin-freezing-technique-could-enhance-future-mrna-vaccines (accessed on 30 July 2024)). For example, a project led by Vaxxas to stabilize mRNA-based vaccines on microarray patches (MAPs), a novel delivery device prioritized by the Vaccine Innovation Prioritisation Strategy (VIPS), is demonstrating thermostability, distribution, and delivery benefits for outbreak as well as routine vaccination.

## 4. The Need for Stability Modelling

A vaccine drug product will experience multiple planned and unplanned changes in environmental conditions as it goes through manufacturing, fill and finish, distribution, and preparation for administration. This includes but is not limited to temperature variations, freeze/thaw cycles, light exposure, and changes in contact materials. Characterization of the degradation profile of a new product requires in-depth understanding of the product properties and the effect of external stress factors on the product quality. This characterization work may necessitate the development or optimization of bespoke assays for the monitoring of degradants, which adds additional complexity and time to the collection of stability data. Hence, establishing the stability profile and defining an expiration date is often a critical path activity in the development lifecycle of vaccine candidates when relying on traditional long-term studies [18]. Furthermore, current regulatory guidance is limited when it comes to a specific description of the mathematical approaches for the evaluation of stability data. Notably, guidelines are proposed in ICH chapters Q1A to Q1E and additionally in Q5C for biological products, with further guidance for vaccines provided by the WHO [19].

Currently, vaccine shelf life is typically granted based on real-time stability data, with the core stability data package described in ICH Q5C. This includes the requirement to submit stability information from at least three batches of the final container product representative of the used manufacturing scale and to provide a minimum of six months’ data at time of submission. There is limited time for real-time stability studies, however, when responding to the outbreak of a novel pathogen. In a rapid response scenario, the total time from outbreak recognition to initial vaccine availability for use may be little more than 3 months [2]. This leaves only 2 months for an initial real-time stability assessment of a new vaccine candidate after accounting for the manufacturing and release of the material (approximately 5 weeks) and testing of stability samples (approximately 1 week). In addition, the approach relies on pooling stability information from pre-clinical material used in toxicology studies, engineering runs, and initial clinical trial supply lots, which may not always be accepted by the health authorities. 

In ICH Q1E, a description of the modelling and stability data evaluation is given, and EMA, FDA, and WHO have published guidelines on stability testing and statistical approaches as well [19,20]. While these documents state that the relationship between an attribute and time can be described by both linear and non-linear functions, it is up to the manufacturer to show that the model is appropriate and to provide sufficient confidence for stability predictions. Additionally, complex biological products have many degradation pathways which are non-linear under accelerated conditions, and a linear regression model cannot predict the change under varying conditions. In the face of biological complexity and the absence of defined mathematical approaches, developers traditionally gathered significant real-time stability data to formulate a shelf-life claim. Collecting such extensive stability evidence may take multiple years, however. Time which is not available in an outbreak scenario.

The aim of stability modelling would be to be able to claim a shelf life allowing widespread distribution (e.g., 12 months at 2–8 °C) based on data that can be generated within the 100-day development timeline. To achieve this level of stability understanding under such a compressed development and manufacturing time, a risk-based approach on shelf-life definition will be required. To achieve this, the principles applied to seasonal flu vaccine production can be leveraged for rapid response vaccines. For seasonal flu vaccines, it may be considered acceptable to extrapolate data from previous years due to tight manufacturing timelines [21]. Similarly, to quickly provide material to vaccination campaigns, prior knowledge of stability data gathered across multiple products of the same manufacturing platform should be utilized. For a new vaccine product, this platform experience is combined with advanced kinetic modelling of short-term stability studies under multiple accelerated conditions to establish an initial shelf-life claim to rapidly make material available for vaccination campaigns. As additional long-term stability information is gathered, the shelf-life claim can be reviewed, and the expiration date updated accordingly.

Table 2 summarizes the information gained from leveraging predictive modelling in vaccine development in a rapid response scenario.

### 4.1. Platform Characterization

In a rapid response scenario, the aim is to provide novel vaccine doses for use within 100 days from the point of outbreak. However, as discussed, this is an ambitious goal which requires a paradigm shift in vaccine development toward preparedness. From a manufacturing perspective, this will only be feasible through the development of vaccine platforms and the adaptation of well-understood prototype vaccines. With sufficient prior experience of the platform, it is expected that large-scale vaccine batches can be manufactured and released in approximately 5 weeks.

Therefore, the development of manufacturing platforms and prototype vaccines is a key element for the success of the 100 Days Mission. This includes the thorough characterization of each vaccine platform and prototype vaccine in the context of stability preparedness. By decoupling the characterization from the rapid response vaccine development, the characterization burden on a new product is significantly reduced. This allows resources to be utilized more efficiently when time is of the essence. Furthermore, the collected platform expertise will ensure new products meet the required stability standard.

Stability studies to collect product and platform stability knowledge are dependent on the stage of development, and different considerations apply to a novel process and/or product vs. a well-established platform process which is used to manufacture materials highly similar to previous products. To maximize the stability information collected, the following points should be taken into account when designing stability studies:

1. Available platform knowledge
  1.1. Historic stability information
    The extent of long-term, accelerated, and stressed stability data which have been collected for the platform and individual products manufactured, including process intermediates.
  1.2. Known impact of critical factors
    Prior knowledge on how modifications of the platform process can impact quality attributes across different products, such as antigen sequence, presence of process- and product-related impurities, differences in concentration, and other variability in physico-chemical properties.
    Understanding of degradation pathways and impacts due to changes in platform processes or modifications to manufacture a new product on the same platform.
    Impact of batch-to-batch variability, manufacturing scale, or manufacturing site on the stability of the final product.
  1.3. Maturity of predictive model
    The availability of a stability model which has been used to accurately predict long-term stability based on accelerated stability studies.
    Understanding of stability study requirements to update an available predictive model for a new product.
  1.4. Product presentation
    Available stability data in different formulations/strengths, as applicable, and when filled in different primary and secondary container combinations. Multidose vials are desirable for outbreak response and require collection of appropriate stability information beforehand.
2. Analytical support
  2.1. The quality of the stability knowledge is highly dependent on the suitability of the analytical methods available for product characterization.
  2.2. Determination of the structure–function relationship of the product in its formulation and under various storage conditions requires fit-for-purpose methods.
  2.3. Potency method is key to monitor vaccine efficacy loss.
    Although platform analytical methods can be adapted for a new product with relative ease, the development of a potency assay is likely on the critical path in a rapid response scenario.
  2.4. Implementation of reference standards
    Appropriate reference materials are critical to monitor analytical performance and product quality.

Investment into stability preparedness and frontloading the generation of platform stability data ultimately brings benefits that go beyond rapid response readiness. By implementing a stability preparedness strategy, the combination of platform stability data and predictive modelling can be evaluated for products outside the pressure of a rapid response scenario. Such prior knowledge ensures the critical elements in stability study design and execution are well understood. This in turn allows for the optimization of stability protocols as well as the predictive models as information on the manufacturing platform is collected. Additionally, the totality of the data gained from stability preparedness may allow discussion and pre-agreement of study protocols with local health authorities.

### 4.2. Predictive Models

In recent years, it has been demonstrated that advanced kinetic models are not only able to successfully predict non-linear regression over prolonged periods. Such predictions are also feasible when based on observations from a relatively short period at multiple temperatures, thus providing an opportunity to significantly shorten the time required to evaluate the stability profile of new products. In addition, kinetic models can be leveraged to assess the impact of temperature fluctuations during storage and transportation. Examples of this approach are given below. A detailed discussion of the mathematical foundation is available in published literature [21,22,23].

Briefly, to develop a kinetic model, an understanding of the degradation rate of the product as a function of time and temperature is required. For this, the stability-indicating attributes need to be identified, and appropriate assays for monitoring those attributes are required. Based on observations of the stability-indicating attributes at various temperatures, it is then possible to determine the equation parameters which best describe the collected dataset. Hence, the equations are an empirical model of the reaction kinetics which allow the prediction of long-term stability based on data from multiple short-term stability studies under different accelerated conditions. To determine the most appropriate model parameters, it is important that observations are performed under conditions which show a change in the stability-indicating attribute at multiple temperatures. Additionally, sufficient data points need to be captured for relevant phenomena such as an initial drop.

Predictive stability modelling has been applied successfully to predict the degradation profile of multiple biological products, including inactivated virus, a protein-based vaccine, emulsion-based adjuvant, and mRNA vaccines:

- In a study by Clénet, the forced degradation of a multivalent inactivated vaccine used a combined approach of advanced kinetics and statistical analysis to describe the loss of antigenicity [24]. Results showed that six months of data under multiple accelerated conditions were sufficient to accurately predict the product stability out to thirty months. In addition, the kinetic model correctly predicted the loss in antigenicity following experimental temperature excursions.
- In another study by Clénet et. al., a kinetic model for a protein-based vaccine has been validated [22]. Following data collection over six months, the model accurately predicted stability out to 24 months. In the same publication, the validation of an autocatalytic-type kinetic model is presented for an oil-in-water adjuvant formulation.
- Castellanos et. al. describe the use of advanced kinetic modelling for a commercial vaccine [25]. Data from an accelerated stability study performed over 6 months were used to establish the model, which then correctly predicted potency loss of the product for up to 3 years.
- For rapid response vaccine manufacturing, mRNA-based products are considered key. Efforts are ongoing to identify relevant stability-indicating attributes, develop kinetic models, and confirm the validity with experimental data, as described by Kis [23]. To date, degradation of mRNA has been confirmed to demonstrate Arrhenius behaviour [26,27]. In addition, the ability to model mRNA vaccine stability using first-order kinetics has been demonstrated [28].

Advanced mathematical models have been shown to accurately predict the shelf life of various biological products, and these data have been used to support new stability claims for vaccines with positive outcomes. Through such predictive models, more stability information is gathered on new products, which, in turn, leads to a better understanding of the stability profile of a new product. Utilizing these data as primary evidence for expiration dating can therefore be considered the state-of-the-art in vaccine development.

Beyond the shelf-life setting, kinetic models can also be used during development to select the most appropriate storage temperature. Another opportunity is the assessment of temperature excursions and their impact on shelf life. The continuous monitoring of residual shelf life through a data logger programmed with an advanced kinetic model has been demonstrated by Roduit et al. [29]. Equipped with data transfer capability and with the appropriate infrastructure available, the implementation of such a data logging device would potentially allow for real-time monitoring of vaccine stocks across the supply chain. It is also feasible to pre-program data loggers with the kinetic model and present the residual shelf life on an integrated display on the data logger. Such a device would negate the need for data connection infrastructure, which may not be available in remote locations.

Additionally, kinetic models can be used to evaluate lot-to-lot differences, the impact of manufacturing scale, or changes in manufacturing site. Furthermore, the variability of the stability profile across products produced with the same manufacturing platform can be compared. With sufficient knowledge of product stability from the platform (across multiple products at various scales and manufacturing sites) it is therefore feasible to leverage this platform stability knowledge and significantly reduce the amount of stability characterization required for new vaccine candidates.

### 4.3. Stability Execution

**Figure 2:**
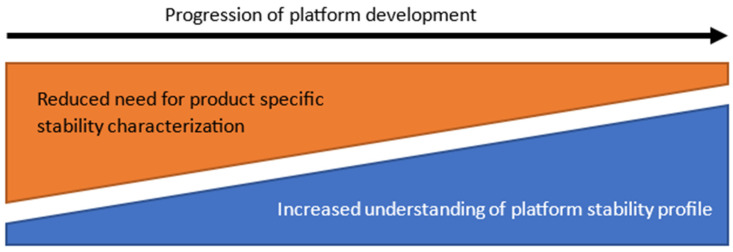
With increased understanding of the stability profile exhibited by products manufactured in a given process, the need for stability characterization is reduced over time.

Combining prior stability knowledge from platform readiness activities with the product-specific data from predicted stability modelling is considered a key enabler for rapid stability assessment in an outbreak scenario. As experience with the manufacturing platform is collected, there will be an increased understanding of parameters which can impact product-specific stability (Figure 2). This knowledge will allow the optimization of stability studies and, ultimately, is expected to lead to a decrease in the product-specific studies needed. Similarly, the use of predictive stability modelling across multiple products of the same manufacturing platform will increase understanding of model selection and the type of studies which will be most suitable to expand the platform- and product-specific stability knowledge space.

Approaches to determine the most appropriate kinetic model should follow established best practices [18,24,30] in a stepwise approach:

1. Stability studies
  Note: Subject to available comparability data and/or prior knowledge, it is feasible to pool stability information from multiple lots, material generated across different clinical phases, and lots produced across more than one manufacturing sites to boost the stability data package which is used in subsequent steps.
  1.1. Stability-indicating attribute and assay selection.
  1.2. Samples are incubated using at least three different temperatures.
  1.3. Periodic testing is performed to acquire at least 20 to 30 experimental data points.
  1.4. Replicates should be favoured over additional time points, and the variability of the analytical method as well as knowledge of the variability structure (in terms of between- and within-run variability) are key variables that need to be considered at this step.
2. Screening kinetic models to fit experimental data
  Run fitting procedures from simple first order to more complex models.
3. Identification of the appropriate model
  The model best describing the observed stability data over all temperatures is identified according to statistical parameters (quality of fit, model comparison score).
4. Determination of prediction accuracy
  4.1. The selected model is used to predict reaction progress under any time/temperature.
  4.2. Prediction accuracy uncertainties are assessed by statistical methods.
5. Verify platform stability
  5.1. Compare the new product model with the available platform stability information.
  5.2. For the new product, confirm a similar trend is observed under accelerated conditions to leverage platform stability information.
  5.3. Claim the shelf life for the new product based on the platform stability data.
6. [OPTIONAL] Model refinement
  With increased understanding of the stability profile and generation of additional data, the model may be refined to better reflect the observed reaction kinetics.

In a rapid response scenario, approximately eight weeks will be available to perform the required stability studies. An exemplar stability study plan is shown in Table 3. For product-specific stability evaluation, the prior stability knowledge and variability of analytical methods need to be considered for the final design of the stability protocol. In general, the selection of time points and accelerated and stressed storage conditions should aim for a degradation larger than the analytical variation and collect stability information from at least four data points at the recommended storage temperature. For products shipped frozen and stored in a refrigerator at the administration site, different models for the frozen and liquid condition need to be developed. While this last scenario can maximize shelf life, it complicates stability assessment as well as monitoring and thus necessitates clear and flexible tracking and labelling systems.

## 5. The Need for Agile Labelling Systems

Rapid development of vaccine candidates to address the COVID-19 pandemic has highlighted the challenge of updating the expiration dates of vaccine lots as new stability evidence is being collected. Historically, the WHO has developed model labels for vials and packaging. This provided guidance on the advised format to manufacturers for many vaccines. Similarly, the WHO has developed a working position for the vial and carton label to unify the labelling requirements supplied through the COVAX facility [31].

In addition, PATH have performed a User Evaluation of COVID-19 Vaccine Labels to unify labelling requirements for vaccines supplied through the Access to COVID-19 Tools Accelerator [32]. Responses to the questionnaire clearly highlighted the desire from end users to have the expiration date and Vaccine Vial Monitor (VVM) included on vials. A VVM is considered best practice, and its absence may lead to hesitancy to use vials for immunization. Similarly, printed expiration dates should be included as soon as feasible, as the absence of this information or handwritten dates on the label after opening are seen as a high risk for administration errors. In addition, the amount of information stated on the vial should be limited to essential information which can easily be identified under low-light conditions. QR codes, barcodes, or website links are considered useful in authenticating the vaccines and obtaining further information. However, such information should not replace key information on the vial, including expiry date, as internet access may not be available in remote areas.

When responding to an outbreak of a novel pathogen, the necessary information to print expiration date and generate a VVM may not be available, however. A situation that is similar to the distribution of COVID-19 vaccines. In the early stages of vaccine supply, vials have been labelled without a printed expiration date [32]. Furthermore, a VVM was not available for new products and was not included initially. Additionally, multiple sites are used to produce vaccine doses, which can lead to differences in vial labels and/or types of packaging, adding to the complexity of labelling. Therefore, job aids have been developed by the WHO to inform health care workers on the importance of maintaining cold chain conditions and how to handle candidate vaccines at the vaccination site in the absence of a VVM [33].

To address updating the expiration date, “dynamic labels” have been applied for the Moderna and Pfizer-BioNTech COVID-19 Vaccine [34,35]. This approach allowed the extension of the expiration date by confirming the latest expiration date of the received material on the product website. Although the approach necessitated internet connectivity and the manual update of the expiration date, it allowed the continued use of manufactured and distributed material as new stability information was collected.

At this point, there is no technical solution which would ensure vial labels contain an expiration date and VVM from the first lot manufactured, unless a platform stability model with a platform VVM is applied. The need for rapid release in an outbreak response is in direct opposition to the time required for stability characterization. Therefore, it is likely that agile labels with a provisional minimal shelf life and without a VVM are required in a potential future rapid response scenario, and mechanisms to update the expiration date manually need to be implemented during vaccine distribution.

Nevertheless, innovations in the real-time tracking of storage temperature can reduce situations in which stable products are discarded due to short-term cold chain breaks. Also, real-time tracking can reduce the risk of vaccines with reduced potency following a temperature excursion being administered. Such systems could allow the verification of the stability profile across the supply chain by continuously collecting temperature data. In the case of temperature excursions, the impact can rapidly be assessed by evaluating the collected monitoring data against the established predictive stability model. At central sites equipped for this complex task, the residual shelf life based on the latest stability data can be calculated, noted on shipment cartons, and transferred to vials manually, prior to last-mile delivery. Though the monitoring is not down to vial level as a VVM provides, such technology could reduce wastage and limit the need for manual look-up of the latest expiration date for individual vaccine lots. It is essential to develop such sophisticated methodologies in a way that also is robust in low-resource settings, aligned with standard vaccination practices, and provides user-friendly and unambiguous information to healthcare workers. 

In addition, additional upfront training of healthcare workers will increase awareness on potential differences in rolling out vaccines for rapid response. This is likely to increase frontline worker acceptability of vials without an expiration date and VVM.

## 6. Towards Stability Preparedness

This Review highlights the key role stability innovations play in the success of the 100 Days Mission. Multiple stability related innovations will be required to achieve a stability readiness which allows the rapid response of vaccine candidates in an outbreak situation and to ensure equitable access to newly developed products. Table 4 provides a summary of the identified topics which require innovation at a scientific/technical level or a review of health authority guidelines to develop robust mechanisms for an outbreak response.

As indicated for various topics in Table 4, alignment with regulatory authorities prior to an outbreak situation is essential for these innovations to have a real impact on vaccine availability. This requires constant dialogue between vaccine and technology developers, health authorities, global health organizations, and other stakeholders. It needs to be recognized that drivers and incentives are different in epidemics and pandemics as compared to business as usual: in non-outbreak situations, there is less need for speed, and the risk/benefit assessment by regulatory authorities will be different. Nevertheless, innovations in the field of thermostable formulations, platform data, prior knowledge, stability modelling, and agile labelling need to be implemented now, to achieve stability preparedness for the next epidemic or pandemic.

## 7. Discussion

Stability preparedness is a key enabler to achieve a response time of 100 days in case of a future outbreak. This Review paper identifies the key innovation areas need to achieve stability preparedness and highlights a range of topics which require further discussion across vaccine developers, industry partners, and public health bodies.

**Figure 3:**
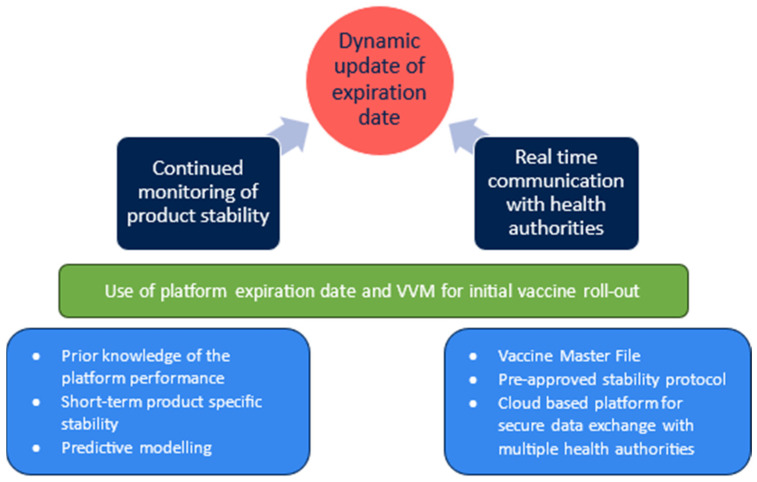
A flexible approach to shelf-life extension is reliant on the continuous generation of product stability data and near real-time exchange of the collected information with health authorities. Generation of platform stability data and the selection of appropriate predictive modelling approaches are the key pillars of rapidly characterizing the stability profile of new products. To initiate quick roll-outs of initial vaccine doses for vaccination campaigns, a platform expiration date can be assigned to new products based on sufficient prior knowledge and with minimal short-term product-specific stability data. The expiration date is then updated in near real time as new data from ongoing product-specific stability studies are being collected. Innovations in how developers can engage with health authorities to rapidly exchange information as it becomes available in the development lifecycle could significantly reduce the response time in vaccine deployment.

Investment into thermostable formulations or product presentations is a key innovation area to address the challenges of distribution associated with thermolabile products (Figure 3). More stable products will ease the burden on infrastructure and, as such, increase equitable access and reduce vaccine wastage due to temperature excursion. Building upon the foundation of thermostable formulations, the generation of platform stability knowledge and development of appropriate predictive models for rapid assessment of new products are key to moving towards stability preparedness.

Although investment in each area individually will support the product development in its own way, linking platform stability data with product-specific stability information through predictive modelling will add considerable value and speed to vaccine development projects where the same manufacturing process is being utilized. The stronger these foundations are for a platform process, the more weight the pillars can carry in terms of predicting the shelf life of new products and, ultimately, allowing the flexible extension of the product expiration date.

Rapid and equitable access to vaccines is possible through a paradigm shift in how development, manufacturing, and distribution in an outbreak scenario are approached. This Review identifies the key topics which need to be addressed to take another step towards preparedness. However, development of a manufacturing platform for thermostable vaccine supporting the 100 Days Mission can only be achieved through collaborative efforts across vaccine developers, industry partners, funding bodies, health authorities, and global health policy makers.

## 8. Conclusions

Stability preparedness is an essential part of pandemic preparedness, to be able to rapidly develop vaccines that can be deployed equitably around the globe. To achieve stability preparedness, innovations need to be advanced and implemented in the fields of thermostable formulations, platform data, prior knowledge, stability modelling, and agile labelling. In addition, alignment between all stakeholders, including vaccine and technology developers, regulatory authorities, and global health organizations, is instrumental.

## Figures and Tables

**Table 1:** Benefits of thermostable vaccines.

Benefit	Description	Impact
Enhanced Global Access	Vaccines with improved thermostability can be distributed in regions lacking ultracold storage facilities.	Ensures equitable access to vaccines, especially in low-income and remote areas.
Reduced Cold Chain Dependency	Stable vaccines reduce the need for ultracold storage and complex logistics.	Simplifies the supply chain and lowers distribution costs.
Minimized Vaccine Waste	Thermostable vaccines are less likely to be compromised by temperature excursions.	Reduces the number of doses lost during transportation and storage.
Extended Shelf Life	Improved stability extends the usable life of vaccines.	Increases the time available for distribution and administration, reducing waste.
Rapid Deployment in Emergencies	Stable vaccines can be rapidly deployed without the need for stringent storage conditions.	Enhances the ability to respond quickly to pandemics and outbreaks.
Lower Financial Burden	Reduced need for specialized storage equipment lowers overall costs.	Makes vaccination programmes more affordable and sustainable.
Environmental Benefits	Less reliance on energy-intensive cold storage reduces greenhouse gas emissions.	Supports global efforts to mitigate climate change.
Increased Public Trust	Reliable vaccines with stable efficacy build confidence in vaccination programmes.	Encourages higher vaccination rates and better public health outcomes.
Facilitates Remote and Outreach Programmes	Stable vaccines are easier to transport to and store in remote areas.	Improves vaccination coverage in hard-to-reach populations.
Innovation in Vaccine Technology	Drives research and development of new formulations and delivery systems.	Leads to overall advancements in vaccine science and technology.

**Table 2:** Benefits of thermostable vaccines and predictive modelling.

Key Point	Description
Environmental Changes	Vaccines face temperature variations, freeze/thaw cycles, light exposure, and contact material changes during manufacturing, distribution, and administration.
Degradation Profile Characterization	Requires in-depth knowledge of product properties and external stress factors, often necessitating bespoke assays for monitoring degradation.
Critical Path Activity	Establishing stability profiles and expiration dates is critical in vaccine development, typically relying on long-term studies.
Regulatory Guidance	Limited specific mathematical approaches in ICH guidelines (Q1A to Q1E, Q5C) and WHO guidelines for evaluating stability data.
Real-time Stability Data	Shelf life is based on real-time stability data from at least three batches, but rapid response scenarios limit available time for these studies.
Rapid Response Scenarios	In outbreaks, the timeline from recognition to vaccine availability is around three months, limiting stability assessment time.
Pooling Stability Information	Initial stability claims may use data from pre-clinical material, engineering runs, and clinical trial lots, though acceptance by health authorities varies.
Modelling and Stability Evaluation	ICH Q1E mentions linear and non-linear functions but lacks detailed methodologies; biological products have complex, non-linear degradation pathways.
Risk-based Approach	A risk-based approach, leveraging seasonal flu vaccine principles, is needed to claim a shelf life within a 100-day development timeline.
Extrapolation of Data	Utilizes data from previous seasonal flu vaccines or similar platform products to quickly establish initial shelf-life claims.
Advanced Kinetic Modelling	Combines platform experience with short-term stability studies under multiple accelerated conditions to establish initial shelf-life claims.
Review and Update of Shelf Life	Shelf-life claims are reviewed and updated with additional long-term stability data to ensure continued vaccine efficacy and safety.

**Table 3:** Study plan to collect stability data within the two months available in a rapid response scenario. Storage conditions and time points should be selected based on the understanding of stability-indicating parameters and the available platform stability data. T0 = study initiation, W = weeks, D = days.

Storage Condition	Example Time Points				
Real storage temperature	T0	1 W	2 W	4 W	8 W
Accelerated 1	0.5 W	1 W	2 W	4 W	8 W
Accelerated 2	1 D	3 D	6 D	12 D	
Stressed	1 D	2 D	3 D		

**Table 4:** Identified innovation areas.

#	Topic	Details	Innovation Needs
1	Development stage of vaccine manufacturing platforms	Platform development ongoing; focus on mRNA due to highest potential to meet rapid response requirement.	Improvement of unit operations or novel technologies to streamline production and release of vaccine candidates.
		Lifecycle management; risk of resetting stability knowledge with significant changes to the product.	Enhanced understanding of how process changes or different product attributes may impact stability profile.
2	Thermostable presentation	Increased stability reduces the risk of stability failure, reduces supply chain pressure, and allows for equitable access due to ease of distribution.	Improvement of product formulations or novel technologies for primary and secondary container closure systems.
3	Stability testing	Pre-approved stability protocols allow rapid generation of additional data to inform expiration dating. However, timelines to notify health authorities and awaiting approval cause delay in applying updated expiration dates to newly manufactured lots.	Identify and agree on a mechanism for health authority notification as a new expiration date is proposed.
		Appropriate analytical methods are required to observe changes in stability-indicating parameters. Product-specific assay development may be on the critical path for initiation of stability studies.	Platform potency assays or surrogate methods to monitor loss of potency in stability studies and throughout the shelf-life period.
4	Multidose vials	Rapid response/large outbreaks may benefit from multidose vials to increase access/speed of delivery and reduce wastage.	Such storage configurations may require specific characterization studies (e.g., container compatibility, homogeneity, microbial growth).
5	Platform stability	Prior platform stability knowledge is used to inform studies of vaccine candidates and provides a baseline for a long-term stability assumption for new vaccine candidates.	Evaluate health authority acceptance and refine conditions which allow the use of platform data.
			Define similarity conditions for the stability profile of a new vaccine candidate when compared to historic data.
6	Predictive modelling	Apply advanced kinetic models to predict stability of new vaccine candidates based on short-term stability data.	Consider revision of ICH Q1E and Q5C to incorporate best practices for stability modelling.
			Develop pre-approved protocols for rapid stability assessment of new vaccine candidates.
7	Real-time monitoring	Utilize real-time tracking devices to monitor storage conditions.	Understand infrastructure requirements and potential hurdles.
			Develop demonstration studies to test the impact the devices can have on cold chain monitoring.
8	Agile label	There are no immediate technical solutions which allow the shipment of vaccine candidates with expiration date and VVM in a rapid response scenario.	With health authorities at national and international level, define a framework for future rapid response supply of vaccine vials through leveraging platform stability and platform VVM.
		Procedures which require look-up of the latest batch expiry date and the manual update of carton and vial information could be standardized for a rapid response scenario and part of healthcare worker training to increase preparedness.	Work with vaccine delivery partners to understand the user requirements and available infrastructure.
9	Platform expiration date and VVM	Development of thermostable vaccine presentations and investments into platform stability characterization may allow the definition of platform stability and generation of a platform VVM—a minimum stability which is understood to be met by all products manufactured within defined process parameters.	Evaluation of scientific, technical, and regulatory feasibility of platform stability which allows shipment of rapid response vaccine lots using platform expiration date and VVM only.
10	Pre-approved “Vaccine DMF”	Submission, review, and discussion of clinical trial applications are a bottleneck in deploying vaccines in an outbreak scenario.	Establish “Vaccine Master File” dossiers at a platform level which are harmonized across geographic regions to speed up revision and review of vaccine candidate applications.
		Segmentation in health authorities across the globe with differing regulations and requirements leads to additional challenges and delays in the product approval process.	Cloud-based platforms for sharing information across multiple health authorities and providing updates in real time as it becomes available in the vaccine development lifecycle can reduce effort in duplicating documentation exchanged with individual health authorities and significantly increase the response time in vaccine deployment.
